# The posttreatment mean apparent diffusion coefficient of primary tumor is superior to pretreatment ADCmean of primary tumor as a predictor of prognosis with cervical cancer

**DOI:** 10.1002/cam4.100

**Published:** 2013-06-16

**Authors:** Keiichiro Nakamura, Satoshi Kajitani, Ikuo Joja, Tomoko Haruma, Chikako Fukushima, Tomoyuki Kusumoto, Noriko Seki, Yuji Hiramatsu

**Affiliations:** 1 Department of Obstetrics and Gynecology, Okayama University Graduate School of Medicine, Dentistry and Pharmaceutical SciencesOkayama, Japan; 2 Medical Radiotechnology, Okayama University Graduate School of Medicine, Dentistry and Pharmaceutical SciencesOkayama, Japan

**Keywords:** Cervical cancer, diffusion-weighted magnetic resonance imaging, mean apparent diffusion coefficient values, predictor for poor prognosis, squamous cell carcinoma

## Abstract

The objective of this study was to investigate the correlation of pretreatment and posttreatment measurements as the mean apparent diffusion coefficient (ADCmean) by diffusion-weighted magnetic resonance imaging (DWI) findings with prognostic factors in patients with squamous cell carcinoma (SCC) of primary cervical cancer. The pretreatment and posttreatment ADCmean of the primary tumor were examined for their correlations with the prognosis in 69 patients with SCC of primary cervical cancer by radiotherapy (RT) with or without concurrent chemotherapy (CCRT). The median disease-free survival (DFS) and overall survival (OS) times of patients were 20.97 and 23.47 months (follow-up periods for DFS and OS: 1–72 and 1–72 months). The DFS and OS rates of patients with low pretreatment and posttreatment ADCmean of the primary tumor were also significantly worse than those of patients exhibiting high pretreatment and posttreatment ADCmean of the primary tumor (DFS;* P *= 0.0130 and *P *< 0.0001, OS;* P *= 0.0010 and *P *< 0.0001). Multivariate analyses showed that low posttreatment ADCmean of the primary tumor was an independent prognostic factor for DFS and OS (*P *< 0.0001 and *P *< 0.0001). The low posttreatment ADCmean of the primary tumor is a useful clinical prognostic biomarker for recurrence and survival in patients with cervical cancer.

The low posttreatment ADCmean of the primary tumor is a useful clinical prognostic biomarker for recurrence and survival in patients with cervical cancer.

## Introduction

Cervical cancer is the second most common gynecological malignancy and the third most common cause of cancer deaths in women worldwide 1. The International Federation of Gynecology and Obstetrics (FIGO) reported a 5-year recurrence rate of 28% for women with cervical cancer 2. The poor prognostic factors for cervical cancer include pelvic lymph node metastasis, parametrial involvement, and tumor volume 3. However, the described parameters are not sufficient to accurately predict the prognosis.

Magnetic resonance imaging (MRI) plays an important role in the diagnosis of cervical cancer. MRI can reveal morphologic characteristics as well as signal intensity characteristics on T1- and T2-weighted images, and contrast-enhanced images. Diffusion-weighted magnetic resonance Imaging (DWI) is a functional imaging technique that analyses differences in the extracellular movement of water protons to discriminate between tissues of varying cellularity 4. DWI has shown its potentially beneficial role for the detection and characterization of malignant tumors. Thus, this technique allows for quantification of diffusion by calculating the apparent diffusion coefficient (ADC) 5. The ADC, a quantitative parameter measured on DWI, has been shown to be useful for the evaluation of solid tumors in the abdomen and pelvis 6–7. It has been suggested that the ADC may provide useful information regarding tumor cellularity, tumor aggressiveness, subtype characterization and cancer treatment response 8–12.

In this study, we investigated the measurements of mean apparent diffusion coefficient (ADCmean) of the primary tumor to evaluate their correlations with the recurrence and survival rates in patients with primary cervical cancer before and after RT or concurrent chemotherapy RT (CCRT).

## Methods

### Study population

The study population consisted of 69 patients with squamous cell carcinoma (SCC) of primary cervical cancer who were treated at the Department of Obstetrics and Gynecology of Okayama University Hospital between April 2006 and February 2013, and those patients who underwent MRI as part of their initial clinical evaluation. The study protocol was approved by the Institutional Review Board of Okayama University Hospital. Informed consent was obtained from all patients. All the patients also underwent routine clinical staging, including their history and a physical examination. The cancers were staged according to the FIGO staging system, and the local disease extents were represented diagrammatically on a tumor staging form. The clinical stages were assessed based on the FIGO staging system as follows: six stage Ib1 cancers; two stage Ib2 cancers; three stage IIa1 cancers; one stage IIa2 cancer; 39 stage IIb cancers; three stage IIIa cancer; 12 stage IIIb cancers; and three stage IVa cancer. The histological cell types were classified according to the WHO classification as follows: all 69 SCCs. The median age was 61.9 years (range, 30.8–89.9 years) (Table 1).

**Table:** Patient and tumor characteristics

Age at diagnosis, *y*	Median, 61.9; range, 30.8–89.9		
	Numbers	%
Stage		
Ib1	6	8.7
Ib2	2	2.9
IIa1	3	4.3
IIa2	1	1.4
IIb	39	56.7
IIIa	3	4.3
IIIb	12	17.4
IVa	3	4.3
Therapy		
CCRT	52	75.4
RT	17	24.6

RT, radiotherapy; CCRT, radiotherapy with concurrent chemotherapy.

### Treatment

In the presence of cervical cancer, patients were advised to undergo RT or CCRT with curative intent. The patients were treated with a combination of external irradiation and intracavitary brachytherapy with curative intent. RT was treated radiotherapy (50 Gy). Overall, 52 patients received concurrent cisplatin chemotherapy (40 mg/m^2^ weekly for six cycles). The remaining 17 patients did not receive concurrent chemotherapy owing to the presence of comorbidities or advanced age (≥75 years) (Table 1).

### MRI acquisition

MRI was performed using a 1.5T MR system (Magnetom Avanto; Siemens, Erlangen, Germany) with a with a six-channel phased-array coil. Routine pelvic MRIs were acquired as follows: axial and sagittal T1-weighted spin echo (SE) (repetition time [TR]/echo time [TE], 600/12 msec; section thickness/intersection gap, 8/0.8 mm, a field of view [FOV], 240–260 × 240–260; matrix size, 320 × 256; number of excitation, 1; acquisition time 1.5 min) images, and axial and sagittal T2-weighted fast SE images (TR/TE, 3000/100 msec, intersection gap, 6/1.5 mm, FOV, 240–260 × 240–260; matrix size, 512 × 256; acquisition time, 2.6 min). Axial DW images were then obtained. Imaging parameters for DW imaging were as follows: TR/TE, 3300/85; flip angle, 90 degrees; number of excitation, 8; matrix size, 128 × 90; band width, 1698 Hz/pixel; section thickness/intersection gap, 6/1.5 mm) using a chemical shift-selective fat suppression technique (SPAIR, spectral adiabatic inversion recovery) and a parallel imaging technique (GRAPPA-2, generalized autocalibrating partially parallel acquisitions-2). The corresponding *b*-values to the diffusion-sensitizing gradient were 0, 50 and 1000 sec/mm^2^. Sagittal and axial contrast-enhanced MR images (TR/TE, 430/11 msec; intersection gap, 8/0.8 mm; FOV, 240–260 × 240–260; matrix size, 320 × 256; acquisition time, 1.5 min) using a fat suppression technique were additionally obtained after the acquisition of DW images.

### Analyses of image findings

The MRI findings of 69 cervical cancers were reviewed with consensus from two radiologists. DWIs were obtained along each of the *x*-, *y*-, and *z*-axes. The ADC value was calculated according to the formula: ADC = (1/[b2–b1]) ln(S2/S1), where S1 and S2 are the signal intensities in the regions of interest (ROIs) obtained by two gradient factors, b2 and b1 (b1 = 0 and b2 = 1000 sec/mm2 for the 1.5 T scanner). The DW images were analyzed by placing ROIs over the tumors on the ADC map images. The ADC values were calculated from the ROIs by dividing the signal intensity by 1000 to obtain the values in terms of ADC × 10^−3^ mm^2^/sec. The ROI placement and ADC calculations were performed for solid portions of the tumors, avoiding any cystic or necrotic parts. The ADCmean were extracted from the manual placement of at least five circular ROIs that encompassed five voxels (approximately 5 mm^2^).

### Assessment of radiation response

The clinical response criteria were defined before the initiation of the study. Patients underwent measurements of ADCmean by MRI at least 6–8 weeks after the end of the RT or CCRT. ADCmean was considered to be an important, objectively measurable criterion for assessing the response to therapy, and therefore the changes in ADCmean were evaluated as both a single prognostic criterion and in combination with other clinical response criteria. Clinical evaluation of the therapy response was accomplished after completion of the therapy.

### Outcome evaluation

Patients had follow-up examinations approximately every 1–2 months for first 6 months, every 3 months for next 2 years, and every 6 months thereafter. The median follow-up for all patients who were alive at the time of last follow-up was 23.47 months (range: 1–72 months).

### Statistical analysis

Statistical analyses were performed using the Mann–Whitney *U*-test for comparisons with controls and one-factor ANOVA followed by Fisher's protected least significant difference test for all pairwise comparisons. Receiver operating characteristic (ROC) curves was generated for pretreatment and posttreatment ADCmean of the primary tumor to determine the cutoff values for predicting recurrence and survival that yielded optimal sensitivity and specificity. The patients were divided into groups based on the pretreatment and posttreatment ADCmean of the primary tumor cutoff values derived from the ROC curves. Disease-free survival (DFS) and overall survival (OS) of the groups were analyzed using the Kaplan–Meier method, and differences between the survival curves were examined using the log-rank test. To assess the correlations between ADCmean and DFS and OS, we performed multivariate analyses using Cox's proportional hazards model. Variables with a *P-*value of <0.05 in the univariate analyses were entered into the multivariate analyses. *P *<* *0.05 was considered to be statistically significant. Analyses were performed using SPSS software version 20.0 (SPSS Inc., Chicago, IL).

## Results

### Patient characteristics

The patient ages, stages, and therapies are listed in Table 1. The median pretreatment ADCmean and posttreatment ADCmean of the primary cervical cancer in the 69 patients were 0.821 × 10^−3^ mm^2^/sec and 1.282 × 10^−3^ mm^2^/sec, with ranges of 0.557–1.100 × 10^−3^ mm^2^/sec and 0.719–1.594 × 10^−3^ mm^2^/sec, respectively.

Table 2 shows the distribution of cases scored for each of the parameters examined according to the clinical characteristics in the overall population. The pretreatment ADCmean of the primary tumor showed significant associations with the FIGO stage (*P *<* *0.001), tumor maximum size (*P *=* *0.002), and parametrial involvement (*P *<* *0.001). The posttreatment ADCmean of the primary tumor was significantly associated with parametrial involvement (*P *=* *0.045) (Mann–Whitney *U*-test, *P *<* *0.05).

**Table:** Associations of the ADCmean with clinical factors in primary cervical cancer

	Pretreatment ADCmean				Posttreatment ADCmean					
Variable	Numbers	Mean ± SE	*P*-value	Mean ± SE	*P*-value
FIGO stage			<0.001		0.056
Ib1–IIa	12	0.919 ± 0.082		1.349 ± 0.113	
IIb–IVa	46	0.800 ± 0.090		1.267 ± 0.189	
Tumor maximum size			0.002		0.075
<4 cm	22	0.873 ± 0.088		1.332 ± 0.139	
≥4 cm	47	0.797 ± 0.095		1.258 ± 0.193	
Parametrial involvement			<0.001		0.045
Negative	13	0.911 ± 0.083		1.348 ± 0.108	
Positive	56	0.800 ± 0.091		1.266 ± 0.190	
Vagina invasion			0.868		0.34
Negative	39	0.823 ± 0.104		1.300 ± 0.182	
Positive	30	0.819 ± 0.093		1.258 ± 0.178	
Pelvic lymph node metastasis			0.143		0.451
Negative	36	0.838 ± 0.088		1.297 ± 0.183	
Positive	33	0.803 ± 0.108		1.264 ± 0.178	

FIGO, International Federation of Gynecology and Obstetrics; ADCmean, mean apparent diffusion coefficient.

### Univariate survival analyses and multivariate analyses

**Figure 1:**
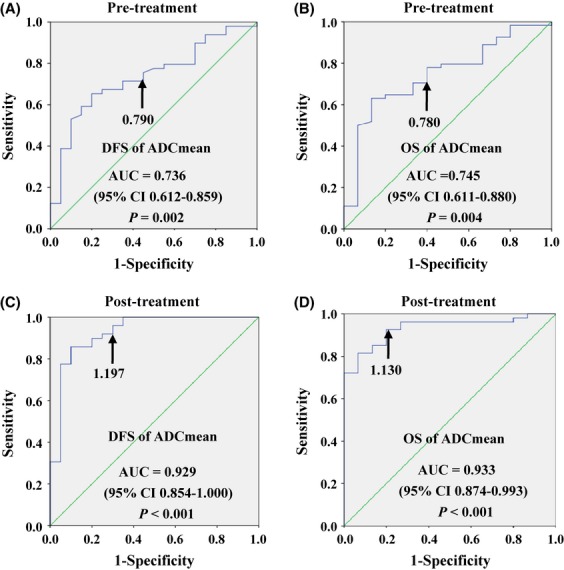
Receiver operating characteristic curves of pretreatment and posttreatment mean apparent diffusion coefficient (ADCmean) of primary tumor for predicting cancer recurrence and survival. (A) Optimal pretreatment ADCmean of the primary tumor for recurrence cutoff values was 0.790 × 10^−3^ mm^2^/sec (area under the curve [AUC] = 0.736, 95% CI 0.612–0.859; *P *=* *0.002). (B) Optimal pretreatment ADCmean of the primary tumor for survival cutoff values was 0.780 × 10^−3 ^mm^2^/sec (AUC = 0.745, 95% CI 0.611–0.880; *P *=* *0.004). (C) Optimal posttreatment ADCmean of the primary tumor for recurrence cutoff values was 1.197 × 10^−3^ mm^2^/sec (AUC = 0.929, 95% CI 0.854–1.000; *P *<* *0.001). (D) Optimal posttreatment ADCmean of the primary tumor for survival cutoff values was 1.130 × 10^−3^ mm^2^/sec (AUC = 0.933, 95% CI 0.874–0.993; *P *<* *0.001).

We used ROC curve analyses to determine the pretreatment and posttreatment ADCmean of the primary tumor of cutoff values to predict recurrence and survival. The analyses identified pretreatment ADCmean of the primary tumor cutoff values of 0.790 × 10^−3^ mm^2^/s for recurrence (area under the curve [AUC] = 0.736, sensitivity 73.2%, specificity 55.0%), and 0.780 × 10^−3^ mm^2^/sec for survival (AUC = 0.745, sensitivity 74.2%, specificity 60.0%), and posttreatment ADCmean of the primary tumor cutoff values of 1.197 × 10^−3^ mm^2^/sec for recurrence (AUC = 0.929, sensitivity 92.7%, specificity 75.0%), and 1.130 × 10^−3^ mm^2^/sec for survival (AUC = 0.933, sensitivity 93.1%, specificity 73.3%) (Fig. 1).

The median DFS and OS times of all patients were 20.97 months and 23.47 months (follow-up periods for DFS and OS: 1–72 and 1–72 months), respectively. Of 19 patients who experienced disease recurrence, 11 had local recurrence only, six had distant metastasis only, and two had both local and distant disease recurrence (Table 3). At the time of last follow-up, 50 patients were alive with no evidence of disease, 15 patients had died of disease, and four patients were alive with disease.

**Table:** 19 patients who experienced disease recurrence

The duration of follow-up, month	Median DFS: 20.97, OS: 23.47
	Range DFS: 1–72, OS: 1–72
Location	Number of metastasis
Local metastasis	11
Cervix	8
Vagina	3
Distant metastasis	6
Mediastinal LN	3
Lung and liver	1
Lung	2
Local + Distant metastasis	2
Cervix + Lung	1
Cervix + Mediastinal LN1	1

LN, lymph node; DFS, disease-free survival OS, overall survival.

**Figure 2:**
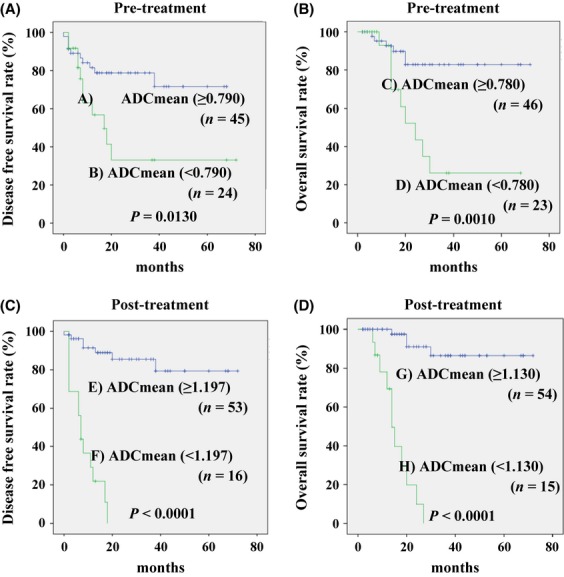
Kaplan–Meier plots for the disease-free survival (DFS) and overall survival (OS) rates of the 69 patients with the assessment of squamous cell carcinoma (SCC) of primary cervical cancer, according to the pretreatment and posttreatment mean apparent diffusion coefficient (ADCmean) of primary tumor. (A) The pretreatment ADCmean of the primary tumor for recurrence (DFS). (A) ADCmean of the primary tumor ≥0.790 (×10^−3^ mm^2^/sec) (*n *=* *45), (B) ADCmean of the primary tumor <0.790 (×10^−3^ mm^2^/sec) (*n *=* *24). (B) The pretreatment ADCmean of the primary tumor for survival (OS). (C) ADCmean of the primary tumor ≥0.780 (×10^−3^ mm^2^/sec) (*n *=* *46), (D) ADCmean of the primary tumor <0.780 (×10^−3^ mm^2^/sec) (*n *=* *23). (C) The posttreatment ADCmean of the primary tumor for recurrence (DFS). (E) ADCmean of the primary tumor ≥1.197 (×10^−3^ mm^2^/sec) (*n *=* *53), (F) ADCmean of the primary tumor <1.197 (×10^−3^ mm^2^/s) (*n *=* *16). (D) The posttreatment ADCmean of the primary tumor for survival (OS). (G) ADCmean of the primary tumor ≥1.130 (×10^−3^ mm^2^/sec) (*n *=* *54), (H) ADCmean of the primary tumor <1.130 (×10^−3^ mm^2^/sec) (*n *=* *15).

Figure 2 shows DFS and OS curves for the 69 patients with cervical cancer, according to the pretreatment and posttreatment ADCmean of the primary tumor. All 69 cervical cancer cases were classified into two categories based on the cutoff values of the pretreatment and posttreatment ADCmean of the primary tumor. The Kaplan–Mayer curves show that the DFS and OS rates of patients exhibiting low pretreatment and posttreatment ADCmean of the primary tumor were significantly worse than those of patients exhibiting high pretreatment and posttreatment ADCmean of the primary tumor (DFS; *P *=* *0.0130 and *P *<* *0.0001, OS; *P *=* *0.0010 and *P *<* *0.0001). Multivariate analyses showed that posttreatment ADCmean of the primary tumor was an independent prognostic factor for both DFS and OS (*P *<* *0.0001 and *P *<* *0.0001). The tumor maximum size was also an independent prognostic factor for and DFS (*P *=* *0.0214) (Table 4).

**Table:** Prognostic factors for disease-free survival selected by Cox's multivariate analysis

	Hazard ratio	95% CI	Cox's test *P*-value
Disease-free survival			
FIGO stage	2.837	0.324–24.807	0.3459
Tumor maximum size	8.733	1.378–55.329	0.0214
Parametrial involvement	0.174	0.016–1.872	0.1491
Vagina invasion	0.9	0.389–2.085	0.8065
Lymph node metastasis	1.648	0.708–3.837	0.2465
Pretreatment of ADCmean	0.459	0.163–1.293	0.1406
Posttreatment of ADCmean	23.504	8.478–65.159	<0.0001
Overall survival			
FIGO stage	0.673	0.162–2.786	0.5838
Tumor maximum size	1.651	0.592–4.602	0.3378
Parametrial involvement	0.604	0.131–2.796	0.5191
Vagina invasion	1.011	0.567–1.804	0.9691
Lymph node metastasis	0.921	0.503–1.685	0.7896
Pretreatment of ADCmean	0.931	0.416–2.104	0.8721
Posttreatment of ADCmean	7.201	3.263–15.895	<0.0001

FIGO, International Federation of Gynecology and Obstetrics; ADCmean, mean apparent diffusion coefficient; CI, confidence interval.

## Discussion

The stage, lymph node metastasis, parametrial involvement, and tumor volume at pretreatment have been described as important prognostic factors on cervical cancer 13–14. However, the described parameters are not sufficient to accurately predict the prognosis. This is the first study to evaluate the pretreatment and posttreatment ADCmean of the primary tumor, and their possible roles in conjunction with clinical factors in patients with cervical cancer.

Quantitative assessment is possible by calculation of the ADC, which can be measured by DWI 15. It has been suspected that the decreased ADC values in malignant tumors may be caused by their increased tissue cellularity or cell density, larger nuclei with more abundant macromolecular proteins and less extracellular space 16–17. ADC measurement can provide useful information for differentiating malignancy from normal cervical tissues. Different reports showed a significantly lower ADC value for cervical cancer (0.75–1.09 × 10^−3^ mm^2^/sec) than that of normal cervical tissues (1.33–2.09 × 10^−3^ mm^2^/sec) 18–19. Our previous study on preoperative assessment of cervical cancers suggested that the lower ADCmean value is correlated with disease recurrence 20. Harry et al. reported that the pretreatment and posttreatment of cervical cancer were examined and the correlations with ADC value, clinical and MR response values in patients before and after 14 days of RT or CCRT. After 14 days of treatment, a significant correlation was found between ADC values and eventual MR response and clinical response 21. Levy et al. reported that posttreatment of cervical cancer in 49 patients were examined with ADC value for cervical cancer after 4–20 weeks of RT. The treatment response was determined based on the histopathological results after RT. The ADC values for complete response of cervical cancer tissues in patients treated with RT were higher than those of residual disease (RD) in patients treated with RT. ADC values could potentially be used to predict and monitor the response of cervical cancer 22.

In this study, we sought to clarify whether the pretreatment and posttreatment ADCmean of the primary tumor was correlated with the clinical characteristics and prognosis in patients with primary cervical cancer by RT or CCRT. We found that pretreatment ADCmean of the primary tumor showed significant associations with the FIGO stage, tumor maximum size, and parametrial involvement. The posttreatment ADCmean of the primary tumor was significantly associated with parametrial involvement.

The purpose was to evaluate whether pretreatment and posttreatment measurements of ADCmean of the primary tumor were associated with recurrence and survival in patients with cervical cancer. This study used ROC curve analyses to determine the optimal cutoff values for predicting recurrence and survival. The pretreatment and posttreatment ADCmean of primary tumor cutoff values were 0.790 × 10^−3^ mm^2^/sec and 1.197 × 10^−3^ mm^2^/sec for recurrence and 0.780 × 10^−3^ mm^2^/sec and 1.130 × 10^−3^ mm^2^/sec for survival. The DFS and OS rates of patients with low pretreatment and posttreatment ADCmean of the primary tumor were also significantly worse than that for high pretreatment and posttreatment ADCmean of the primary tumor. Multivariate analyses showed that posttreatment ADCmean of the primary tumor was an independent prognostic factor for DFS and OS in our study population. Interestingly, posttreatment ADCmean of the primary tumor is superior to pretreatment ADCmean of the primary tumor in both recurrence and survival prediction with cervical cancer.

We acknowledge that there are some limitations in our study. First, the number of patients was relatively small. Second, the duration of follow-up was relatively short. A larger number of patients and long-term follow-up would support the strength of our data, and further confirmation by a prospective trial could reinforce our findings.

Our findings provide evidence that posttreatment low ADCmean of the primary tumor is a useful clinical prognostic biomarker for recurrence and survival in patients with cervical cancer.

## Conflict of Interest

None declared.
